# Porous Polyethylene Coated with Functionalized Hydroxyapatite Particles as a Bone Reconstruction Material

**DOI:** 10.3390/ma11040521

**Published:** 2018-03-29

**Authors:** H. Fouad, Randa AlFotawi, Othman Y. Alothman, Basheer A. Alshammari, Musaad Alfayez, Mohamed Hashem, Amer Mahmood

**Affiliations:** 1 Applied Medical Science Department, Community College, King Saud University, Riyadh 11437, Saudi Arabia; 2 Department of Biomedical Engineering, Faculty of Engineering, Helwan University, Helwan 11792, Egypt; 3 Maxillofacial Surgery Department, Dental Faculty, King Saud University, Riyadh 11545, Saudi Arabia; ralfotawei@ksu.edu.sa; 4 Chemical Engineering Department, King Saud University, Riyadh 11421, Saudi Arabia; othman@ksu.edu.sa; 5 Deanship of Graduate Studies, Saudi Electronic University, Riyadh 11637, Saudi Arabia; 6 Material Science Research Institute, King Abdulaziz City for Science and Technology (KACST), Riyadh 11442, Saudi Arabia; b_shammari@hotmail.com; 7 Stem Cell Unit, Department of Anatomy, College of Medicine, King Saud University, Riyadh 11461, Saudi Arabia; Alfayez@ksu.edu.sa (M.A.); amer_dk@yahoo.com (A.M.); 8 Dental Health Department, College of Applied Medical Sciences, King Saud University, Riyadh 11437, Saudi Arabia; omer_hh@yahoo.com

**Keywords:** porous polyethylene, hydroxyapatite, mesenchymal stem cells

## Abstract

In this study, porous polyethylene scaffolds were examined as bone substitutes in vitro and in vivo in critical-sized calvarial bone defects in transgenic Sprague-Dawley rats. A microscopic examination revealed that the pores appeared to be interconnected across the material, making them suitable for cell growth. The creep recovery behavior of porous polyethylene at different loads indicated that the creep strain had two main portions. In both portions, strain increased with increased applied load and temperature. In terms of the thermographic behavior of the material, remarkable changes in melting temperature and heat fusion were revealed with increased the heating rates. The tensile strength results showed that the material was sensitive to the strain rate and that there was adequate mechanical strength to support cell growth. The in vitro cell culture results showed that human bone marrow mesenchymal stem cells attached to the porous polyethylene scaffold. Calcium sulfate–hydroxyapatite (CS–HA) coating of the scaffold not only improved attachment but also increased the proliferation of human bone marrow mesenchymal stem cells. In vivo, histological analysis showed that the study groups had active bone remodeling at the border of the defect. Bone regeneration at the border was also evident, which confirmed that the polyethylene acted as an osteoconductive bone graft. Furthermore, bone formation inside the pores of the coated polyethylene was also noted, which would enhance the process of osteointegration.

## 1. Introduction

Bone augmentation is used in a wide range of clinical surgeries. Although most bone fractures heal normally without intervention, some large bone defects leave remnant deformation, requiring further intervention. Over the past 50 years, a wide variety of alternatives to natural bones have been investigated, such as biomaterials that can be used for the treatment of natural bone defects or damaged and traumatized bone tissue, as substitutes for allogeneic or autologous bone material [1,2,3,4].

An ideal bone replacement material should possess a number of important characteristics, including non-toxicity, ease of adaptation and handling, a small degree of resorption, biocompatibility, durability, capacity to allow the ingrowth of original tissues, early integration, and non-conductivity. The material should also be produced, treated, and available at a reasonable cost [5,6,7].

Bone substitutes are produced using a variety of materials, including polymers, ceramics, and metals and their composites. Polymers are the most common among these materials because of their excellent design flexibility and capacity to be molded into a desired shape depending on the bone defect, in addition to their biodegradability. Therefore, polymeric materials have become attractive materials for these applications. In contrast, most metals have limited processability and lack degradability. Thus, polymers have received extensive attention and are widely considered over other materials in bone tissue engineering research [1,4,7].

For bone tissue engineering, various types of polymeric materials have been used. These can be categorized as natural/biological polymers and synthetic polymers. Synthetic polymers can be processed using various techniques and procedures to fabricate three-dimensional (3D) substrate scaffolds with different surface characteristics and porosities. Moreover, the advantage of synthetic polymers over natural polymers is that they can be produced in a large-scale manner with controlled mechanical properties and degradation rates. Therefore, synthetic biodegradable polymers have been extensively used as scaffolds for bone tissue engineering. The scaffold properties need to be appropriate and the material cannot break down during treatment or during the patient’s normal activities [1,8].

The most popular synthetic polymers for bone tissue engineering are highly porous polymer matrices, which play an important role in facilitating the attachment of cells and the spread, propagation, and formation of new tissue/fluids around the bone defects. Polyethylene (PE), polyurethane, polyetheretherketone, and polysulfone are all biocompatible polymers that are stable in the body, of which PE is the most popular [9].

Porous PE and unresolvable polymers are biocompatible and have been widely used in bone reconstruction for the past two decades. These synthetic polymers have an average pore size of ~100 μm, with a pore volume of ~50%. The architecture of such pores allows the in-growth of new tissue and the incorporation of surrounding tissue with bone [10,11,12,13,14,15,16].

Critical-sized defects in the calvarial bone of rats have been used as a model to test the biocompatibility, osteogenicity, and osteoconductivity of different biomaterials [17,18,19,20]. According to Curzio et al. [6], many studies have concluded that the use of porous PE implants provides excellent results in bone augmentation and reconstruction. In addition, the authors reviewed the results of 347 porous PE implants in 239 patients over 15 years. The implants were placed to treat a variety of craniomaxillofacial deficits. They reported that 94% of 239 consecutive patients had a postoperative course without complications. The most common complications associated with the use of porous PE implants included exposure, migration, infection, fistula formation, cyst-formation, restrictive strabismus, and hemorrhage [14,21].

Despite the aforementioned reports of porous PE as an implant material, detailed information regarding the indications, results, and complications associated with this material is lacking. To the best of our knowledge, few studies have dealt with the viscoelastic characteristics of porous PE implants. Furthermore, we fabricated calcium sulfate–hydroxylapatite (CS–HA)-coated PE and assessed its characteristics in vitro and in vivo. This study focusses on the mechanical and viscoelastic characteristics of these implants and their biological effects when implanted at orthotropic sites.

## 2. Results and Discussion

### 2.1. Morphological Analysis

**Figure 1:**
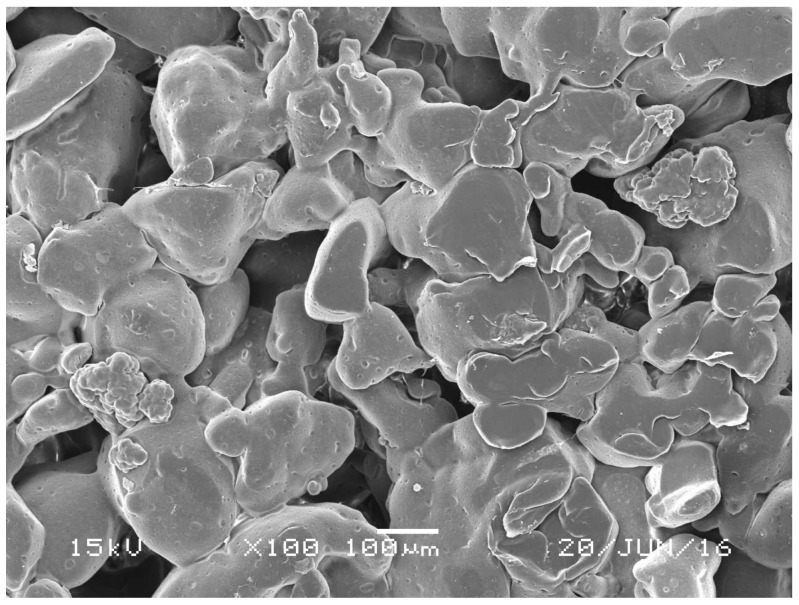
Scanning electron microscopic images of the porous polyethylene. Surface scale bar = 100 μm.

The morphology of porous PE was examined using a scanning electron microscope (SEM), and the images were acquired at 100× magnification, as shown in Figure 1. The pores were distributed and interconnected across the PE matrix. The average pore size ranged from 100 to 400 μm, as specified by the manufacturer. However, a pore size of 100–300 micro with high porosity has been shown to facilitate cell infiltration, bone growth, and internal mineralization [14].

### 2.2. Thermographic Behavior at Different Rates of Heating 

**Figure 2:**
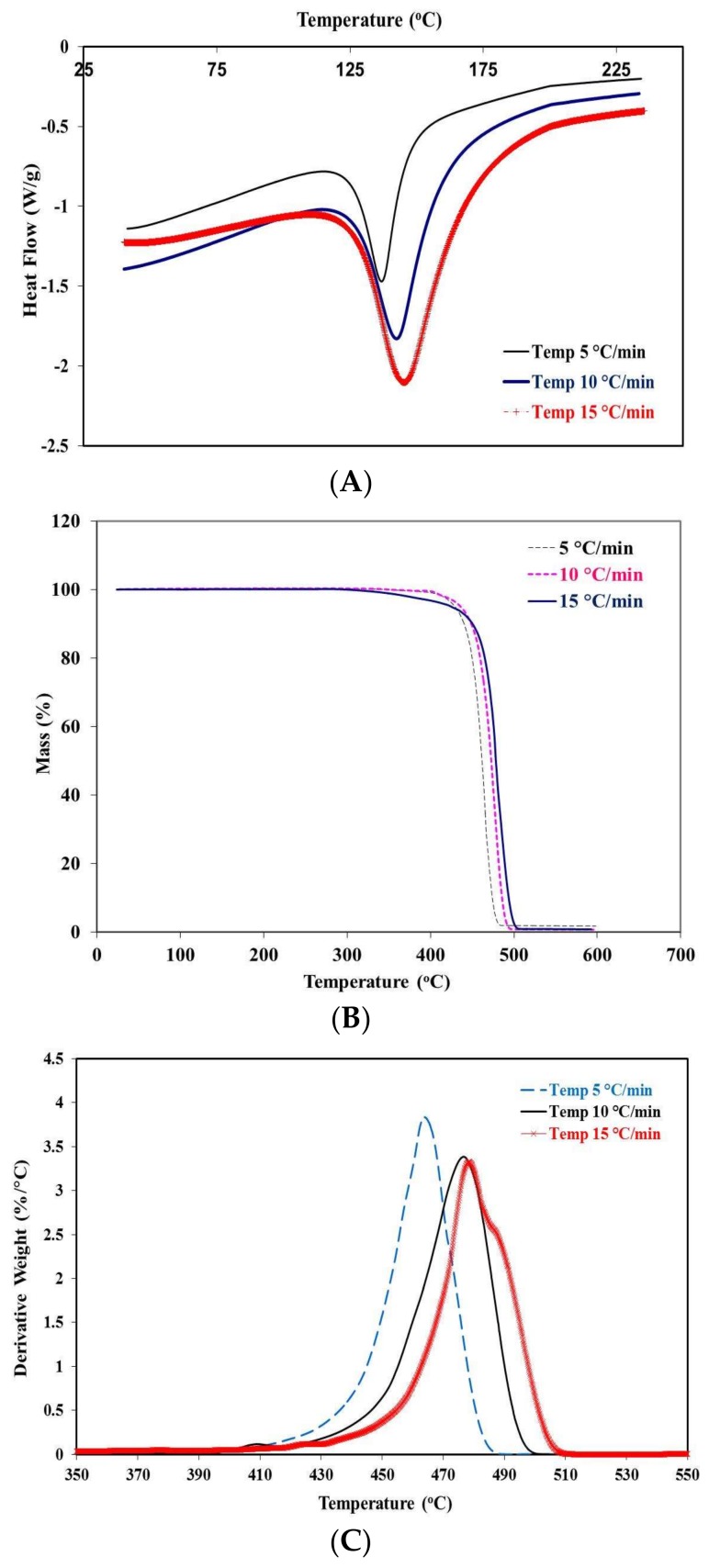
(**A**) Differential scanning calorimetry (DSC); (**B**,**C**) thermogravimetric analysis (TGA) results for porous polyethylene (PE) at different heating rates.

Figure 2 shows the thermal behavior on first heating of porous PE at different heating rates (5 °C, 10 °C, 15 °C). As can be seen in the figure, some changes in the thermal behavior of the PE can be observed. The melting temperature (T_m_) increased from 135 to 142.5 and 145 °C when the heating rate increased from 5 to 10 and 15 °C, respectively. Furthermore, the melting peaks became broader as the heating rate increased (Figure 2A). This change in the thermal behavior of PE can be attributed to the thermal resistance of the polymer, leading to a temperature gradient across the sample [22,23]. Therefore, as the heating rate increased, heat transfer time decreased, resulting in a thermal lag [24,25]. A similar phenomenon was observed for the degradation behavior and thermal stability of porous PE, as shown in Figure 2B,C. The thermogravimetric analysis (TGA) results show that the porous PE lost 10% and 90% of its weight at 390 and 460 °C, respectively, at a 5 °C/min heating rate. The effects of different heating rates on the degradation temperature are clearly seen in Figure 2C. The apparent thermal stability of porous PE increased as the heating rate increased. The temperature for full weight loss increased from 463 to 479 °C when the heating rate increased from 5 to 15 °C. As stated above, the poor thermal conductivity of the polymer hinders it from reaching its expected temperature [22,23].

### 2.3. Creep Recovery Behavior 

**Figure 3:**
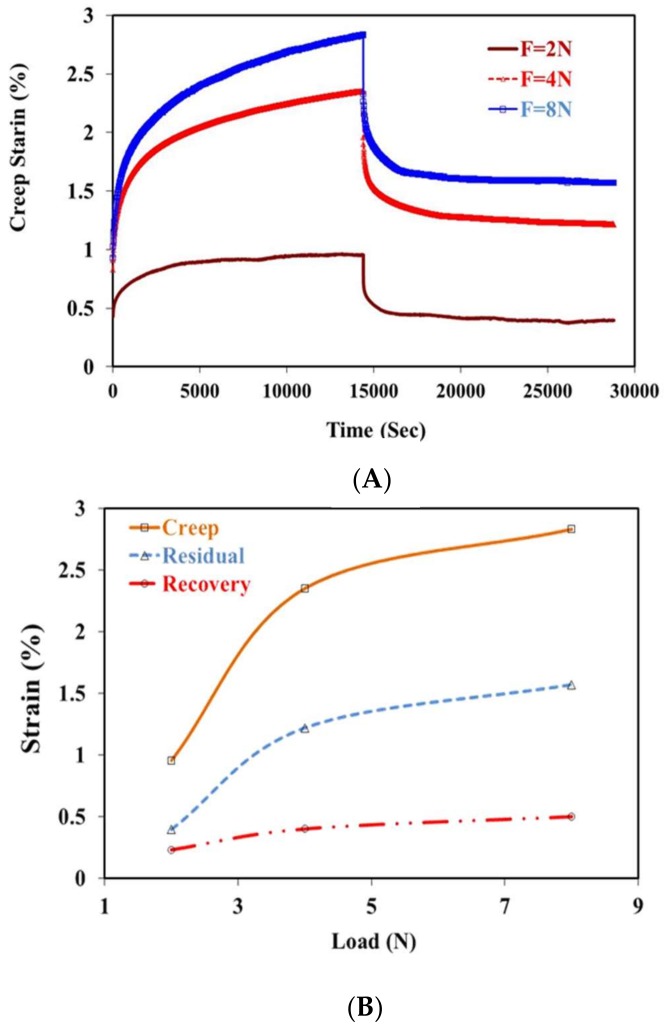
Effects of load on the creep behavior of porous polyethylene.

Figure 3A,B shows the creep recovery behavior of porous PE at different loads for a period of 4 h of loading and 4 h of unloading. The results revealed that the creep strain during the test could be divided into two main portions. The first portion showed an initial increase with load early on. The second part showed a smaller increase with time. In both portions, strain increased with increasing load, as shown in Figure 3. The creep strain increased from 0.956% to 2.35% and 2.83% when the applied load increased from 2 to 4 and 8 N, respectively. The increase in the initial and second portions of strain with increasing load was related to the resultant increase in stress in the specimens. After removing the load, the resultant strain recovered and some residual strain remained. The recovery and residual strains were proportionate to the applied load, as shown in Figure 3B. This recovery strain was also proportionate to the total strain just before removing the load. After 4 h of unloading, the measured residual strain was 0.231%, 0.4%, and 0.5% at loads of 2, 4, and 8 N, respectively. Furthermore, the residual strain was proportionate to the applied load and the total creep strain. Similar results have been reported previously [26,27,28], where the creep strains increased with increasing load.

**Figure 4:**
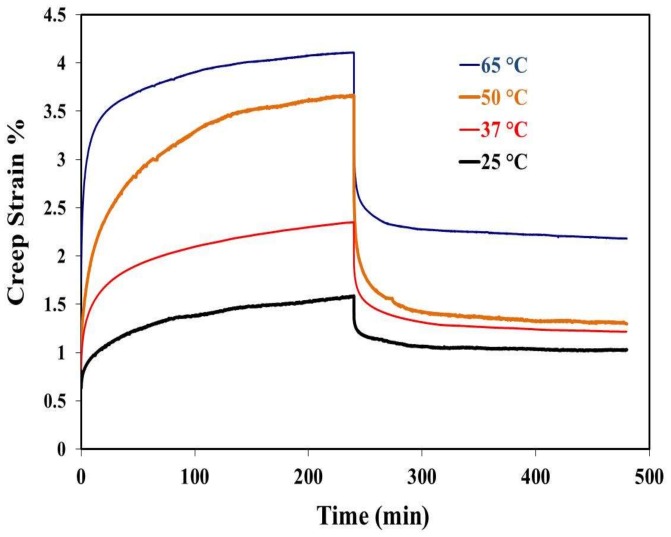
Effects of temperature on the creep behavior of porous polyethylene.

The effect of temperature on the creep recovery strain of porous PE is shown in Figure 4. The porous PE specimens were tested under 4 N and different temperature ranges, as indicated in Figure 4. The results showed that creep and recovery strains increased with increasing temperature. For example, the creep strain increased by 53%, 104%, and 161% when the testing temperature increased from 25 to 37, 50, and 65 °C, respectively. Furthermore, the remaining residual strain in the tested material 4 h after removing the load increased from 1.03% to 1.2%, 1.3%, and 2.2% because of the increase in the testing temperature from 25 to 37, 50, and 65 °C, respectively. This increase in strain represents the greater ease of movement of polymer chains at elevated temperatures.

### 2.4. Relaxation Behavior

**Figure 5:**
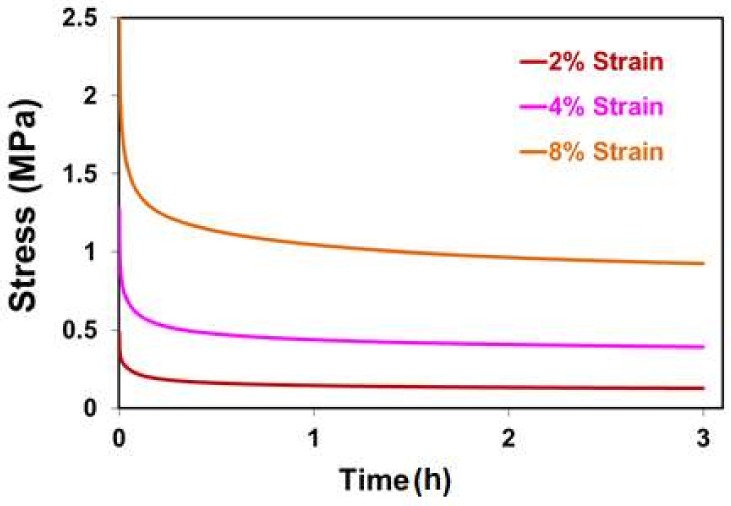
Relaxation behavior of porous polyethylene at different initial strains.

The relaxation behavior of porous PE at 37 °C and +2%, 4%, and 8% is shown in Figure 5. The relaxation of stress was measured at 3 h from initial loading. The results indicated that the porous PE responded with stress values relative to the applied initial strain. The results showed that the stress decreased dramatically at the first hour and nearly reached a constant value at the end of the test for all testing conditions. Similar results were also reported in previous studies, in which the stress displayed the same trend in different testing conditions [28].

### 2.5. Tensile Test Results

The tensile test results of porous PE were obtained at three different cross-head speeds to investigate the effects of loading rate (strain rate) on the behavior of the tested material. The tensile data were obtained as modulus, ultimate strength, and fracture strength values. Table 1 shows the variation of these properties with different cross-head speeds. The results demonstrate the strain rate sensitivity of the tested material; the properties increase with cross-head speed. Similar results were also obtained for non-porous PE in prior studies [26].

### 2.6. Cell Culture Results

#### 2.6.1. Treatment of Porous PE Scaffolds: Composition of the Coating Material

**Figure 6:**
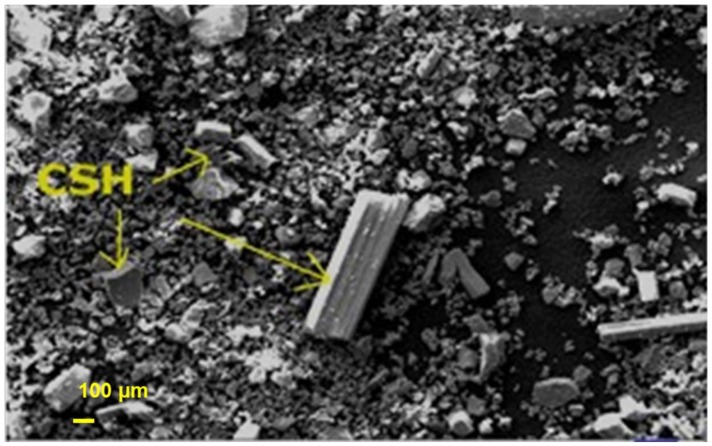
Microradiograph for scanning electron microscopy of calcium sulfate hydrate–hydroxyapatite powder showing calcium sulfate hydrate in rectangular and rod-like shapes (arrows) and smaller particles of hydroxyapatite. Scale bar = 100 μm. calcium sulfate hydrate.

**Figure 7:**
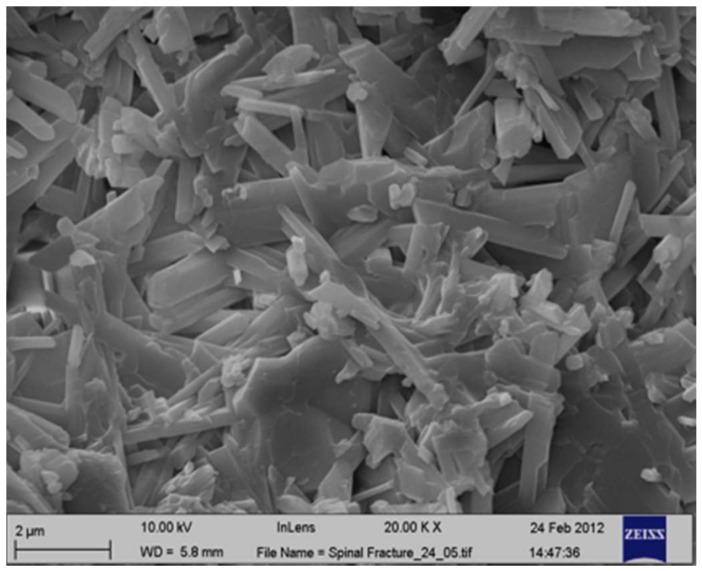
Microradiograph for scanning electron microscopy of calcium sulfate hydrate–hydroxyapatite, which attained compact structures of well-interconnected crystals. The hydroxyapatite crystals are shorter than the calcium sulfate hydrate crystals. Scale bar = 2 μm.

The coating material used in this experiment was formed by combining 60% α-calcium sulfate hydrate (α-CSH) and 40% hydroxyapatite (HA) in powdered form (Figure 6). The liquid phase was an iodine-based, water-soluble, non-ionic radio-opacity enhancing component. When the powder was mixed with the liquid phase, an easily injectable paste was formed. The concentration of the liquid phase had a 180 mg/mL relative concentration. When the material set, it attained compact structures of well-interconnected crystals (Figure 7).

The material is metastable below 40 °C under atmospheric pressure and will hydrate in contact with H_2_O to become calcium sulfate dihydrate (CSD):

CaSO_4_·1/2 H_2_O + 3/2 H_2_O → CaSO_4_·2H_2_O

The long and flat crystals of CSD measured approximately 4–6 μm and spread in every direction, resulting in a matrix of well-organized crystals. The formed cement was not macroporous but rather microporous, with the average size of micropores being 5 μm. Nilsson M. et al. previously quantified the characterization of the pore size [29].

The compressive strength of the CSD–HA scaffold depends on the amount of water added, the morphology of HA, the HA content, and the amount of accelerator used. This value was previously estimated, based on the morphology of HA and it was found to be (31 ± 6.1) MPa [29,30].

**Figure 8:**
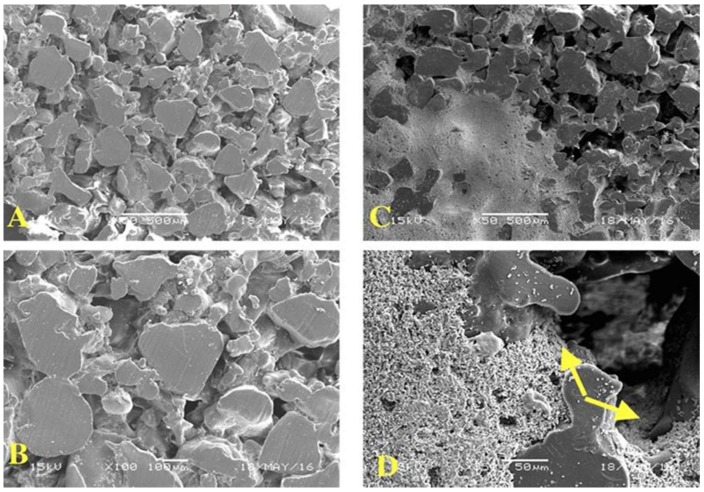
Microradiograph for scanning electron microscopy of porous polyethylene. (**A**,**B**) show the cut section of non-coated porous polyethylene; the variation in pore size is noted to vary within the range of 50–400 μm. (**A**,**B**) scale bars = 500 and 100 μm. (**C**,**D**) show the coated porous polyethylene.

**Figure 9:**
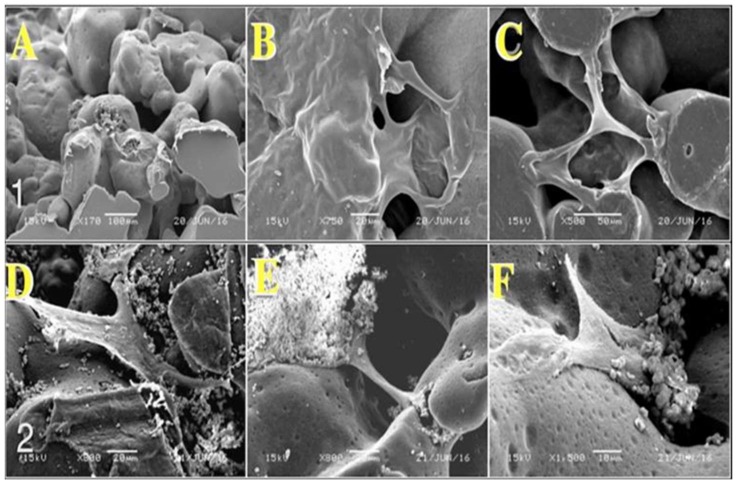
Micrograph of porous polyethylene three days after cell seeding. The first row represents the cell culture on uncoated porous polyethylene discs; cells are mainly found toward the edge of the discs (**A**). Cells are also found crossing the scaffold’s pores (**A**–**C**). Scale bars = 100, 20, and 50 μm. (**D**–**F**) show cells with the typical morphology of stromal cells (polygonal or fusiform). The cells are intermingled with coating material’s crystals, and crystalline apatite on the surface of material can be seen. Cells migrated inside the pores of porous polyethylene. Scale bars = 20, 20, and 10 μm.

An assessment of the cement microstructure using an SEM was performed after the sample coating and the microstructure of CS–HA was found to be compact and to penetrate the pores of porous PE, with HA particles integrated inside the porous PE scaffold with an even distribution (Figure 8B,D). It has been found that the CS/HA ratio favors the spontaneous precipitation of crystalline apatite on the surface of the material, often with complete setting of the material in vivo [30]. This layer of apatite enhances direct contact between the implant and bone (Figure 9D).

#### 2.6.2. Morphological Observation of Stromal Cell Attachment

After three days of culturing hBMSCs-CL1 (CL1) cells, most of the cultured cells adhered and were clearly observable on the CS–HA scaffold surfaces (Figure 9D–F). We found that the cells proliferated better in some areas than in others, and in these areas the cells formed aggregates. Cells cultured on CS–HA measured 60–160 μm in length and were polygonal or fusiform (Figure 9D–F). Furthermore, the cells on the CS–HA scaffold were accompanied by filamentous fibers formed on the surface; we believe that this confirms the presence of extracellular matrix formation (Figure 9D). Furthermore, the cells were intermingled with the coating material’s crystals (Figure 9D–F). In the case of uncoated porous PE, cells were at the periphery of the discs (Figure 9A). Although cells reached the desired size and shape, they were unevenly distributed throughout the examined surfaces (Figure 9B,C).

#### 2.6.3. Viability of CL1 Cells on Porous PE Scaffolds

**Figure 10:**
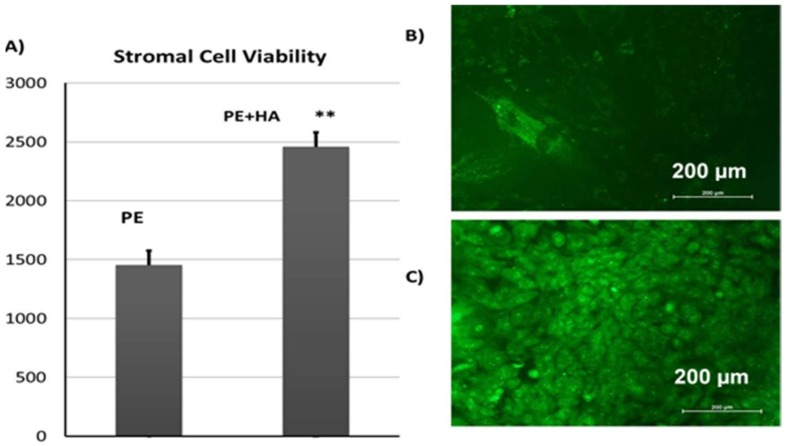
(**A**) AlamarBlue^®^ assay of CL1 cells cultured on porous polyethylene and polyethylene + hydroxylapatite scaffolds; (**B**) CL1 cells grown on a polyethylene scaffold; (**C**) CL2 cells grown on polyethylene + hydroxylapatite scaffolds stained with acridine orange, which stains all nucleated cells green. The magnification used was 10×.

We cultured CL1 cells on the porous PE scaffold with or without HA and assessed cell attachment. Quantitative assays clearly showed that the attachment was significantly better (1.7-fold) when the porous PE scaffold was coated with HA (Figure 10A). This was confirmed by staining the cells with green fluorescent dye (acridine orange stain). This revealed that more cells were attached to the porous PE + HA scaffold than to the porous PE alone (Figure 10B,C).

Many previous studies have investigated the biological fixation of implant materials, and several biomaterials have been used to enhance the process of osteointegration between the prosthetic component and host bone. HA and calcium phosphate (CaP) are examples of coating materials that have been used to coat scaffolds. In this study, a CS–HA composite was used as a coating material for PE discs to enhance the process of osteointegration and minimize the infection rate due to inadequate implant fixation [14].

CS is a recognized bone substitute material. Previously, it has been shown that CSD stimulates the osteogenic differentiation of rBMSCs in vitro [30]. In contrast, CSD has a very high reabsorption rate. The advantage of this high reabsorption rate is the enhancement of pore formation and subsequent angiogenesis and bone ingrowth in vivo [31]. In addition, during bone regeneration, a variety of mechanisms have been proposed for the role of CS, including osteoconduction and/or osteoinduction [32].

The other component of the coating material was 40% HA. HA has a similar chemical composition to the mineral phase of bone. However, it is stoichiometric and has no ionic substitution, like biological apatite. The delay in the reabsorption of HA makes this material suitable for naturally reconstructing the bone. Therefore, the presence of HA particles provides osteoconductivity, promotes integration with the newly formed bone, and ensures sufficient strength after the early healing phase. Interestingly, when the composite bioceramic material CS–HA was tested, the Ca/P ratio of the precipitate was similar to that of the bone mineral. This was reported to stimulate bone ingrowth in vivo. More rapid bone contact was obtained and surfaces were treated with bone-like apatite [29].

Our novel concept is to use an alloplastic material, PE, coated with composite bioceramic CS–HA as a bone graft material to overcome the shortcomings of clinical use of PE bone grafts for augmentation in the craniofacial region [14]. We investigated this novel concept in vitro. A comprehensive assessment of this biomaterial was performed, including cell viability, proliferation rate, cell adhesion, and molecular testing.

Qualitatively, SEM was used for the assessment of the viability of hBMSCs by seeding the cells directly onto the material. SEM evaluation showed that a large number of cells were adherent. A greater degree of hBMSC proliferation was attained with the characteristic morphology of hBMSCs on the surface of the coated polymer vs. the uncoated PE. A comparable SEM discovery has been demonstrated with MSCs cultured on the surfaces of HA/polyamide constructs. This observation revealed significantly greater proliferation of osteoblastic-like cells on coated PE discs than on uncoated PE. On the basis of this finding, it was concluded that the dip-coated disc promoted the differentiation and expression of osteogenic cells and that bone formation would proceed faster on the coated PE discs.

### 2.7. In Vivo Experiments

#### 2.7.1. Gross Clinical Examination

**Figure 11:**
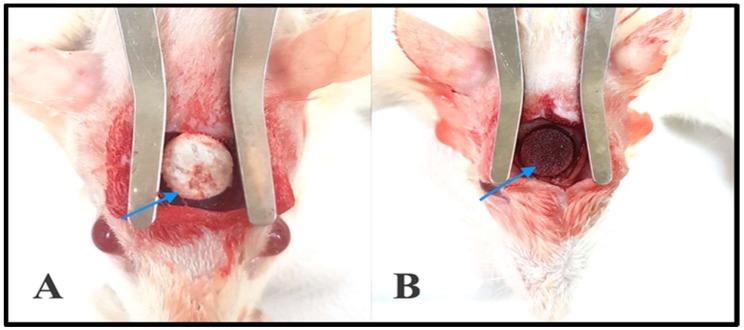
Photographs showing the surgical defects at the rat calvarium. (**A**) Shows the coated porous polyethylene disc in situ; (**B**) Shows the untreated polyethylene disc in situ.

The harvested bone with PE discs showed that a white membrane-like fibrous tissue covered the defect area in both experimental groups (Figure 11). PE discs were in place (at the created defect). The coated samples showed bone growth from the bone border of the defect (i.e., bridging the gap). There were no signs of infection (Figure 11A,B).

#### 2.7.2. Cone Beam Computed Tomography (CBCT)

**Figure 12:**
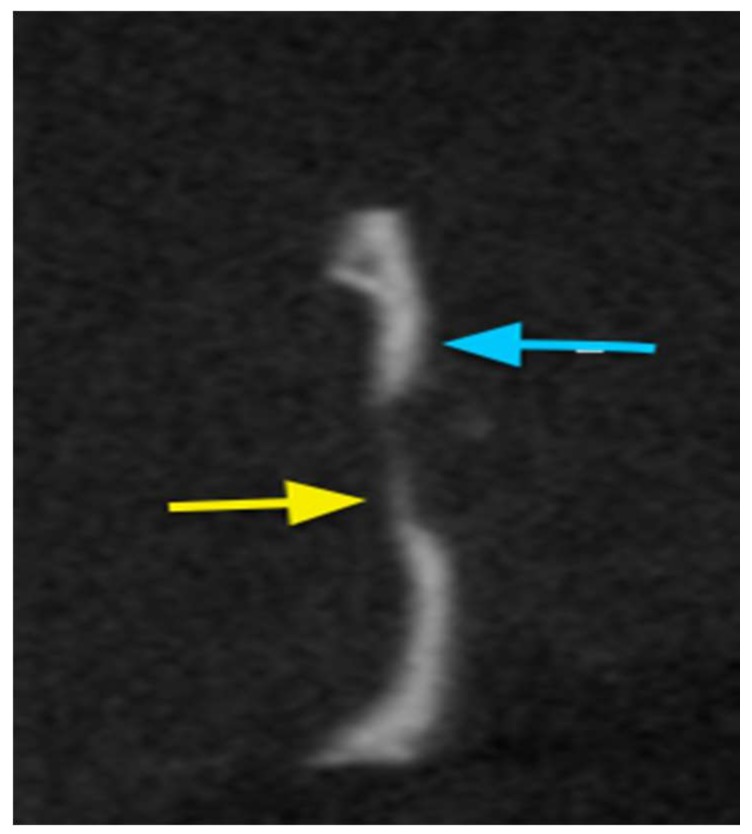
Sagittal view for cone beam computed tomography (CBCT) for the coated PE disc in situ after three months. The area of bone regeneration is shown by the yellow arrow, while the area of native bone is shown by the blue arrow. The area of the PE graft appears radiolucent.

Coronal and axial section cuts were retrieved for each sample. An area of radio-opaque tissue was detected from the border of the defect (interface) with the coated PE. In the case of uncoated PE, the area of interface appeared as a radiolucent area. PE discs appeared as a completely radiolucent area (Figure 12).

#### 2.7.3. Histological Analysis

**Figure 13:**
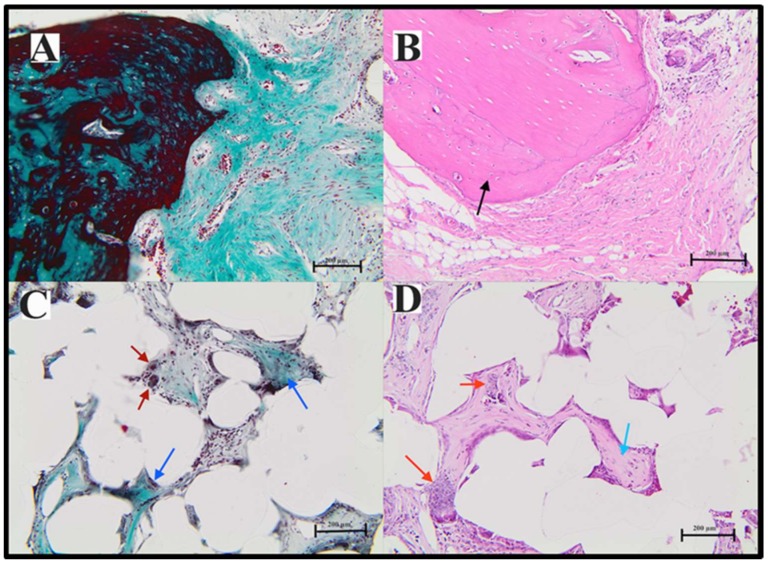
Photomicrographs of decalcified sections stained with hematoxylin and eosin (right) and Masson’s trichrome stain (left) demonstrating the area of bone regeneration at the surgical defect using coated porous polyethylene. (**A**,**B**) are sections through the area at the interface between the bone border of the defect and the implanted coated polyethylene. The bone shows active remodeling, and the connective tissue next to the bone is highly rich with collagen (green) and osteoblasts and preosteoblast-like cells. (**C**,**D**) show sections from the center of the scaffold; the interpore spaces are full of connective tissue that turned into bone and osteoid (**C**,**D** blue arrows). The presence of hydroxylapatite is noted (**D**, red arrow). There are also giant cells seen mainly in this section (**C**, red arrow).

**Figure 14:**
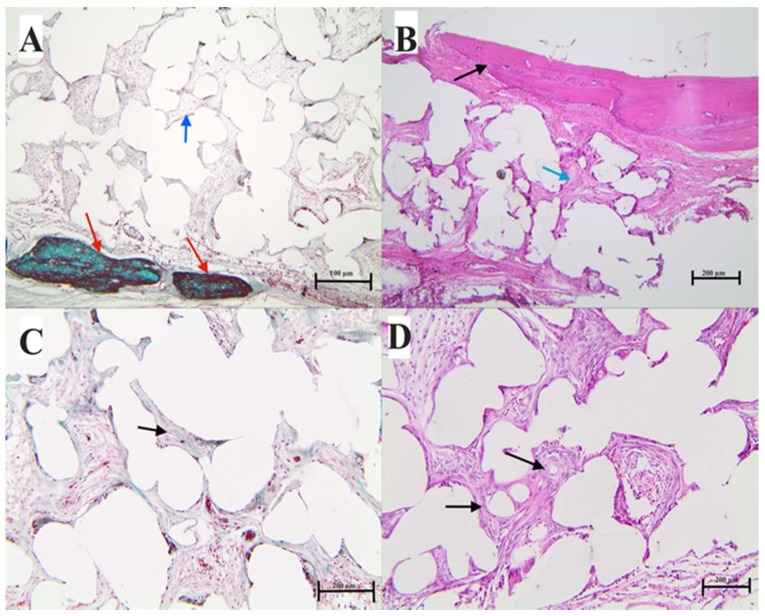
H&E and Masson’s trichrome staining for non-coated polyethylene disks. Decalcified sections were stained with hematoxylin and eosin (right) and Masson’s trichrome stain (left), demonstrating the area of bone regeneration at the surgical defect using plain polyethylene. (**A**,**B**) are sections through the area of the peripheral bone defect and the implanted plain non-coated polyethylene. (**B**) Black arrow shows newly generated bone with evidence of active remodeling. The connective tissue is next to the bone, with osteoblastic and pre-osteoblast like cells (see blue arrows). (**C**,**D**) are sections from the center of the scaffold; the interpore spaces are full of connective tissue that turned into bone in the osteons (**C**,**D** black arrow).

An examination of decalcified sections confirmed that the magnitude of bone regeneration in the defect varied across sections in the different experimental groups (Figure 13 and Figure 14). For both PE groups, the area of bone interface with coated discs showed a highly remodeled border in the defect and a layer of loose connective tissue that was highly cellular with a cell population that consisted of osteoblasts and pre-osteoblasts next to the remodeled bony borders (Figure 14A,B). There was evidence of bone regeneration along the border of the defect in both groups. More interestingly, mature osteons and marrow caves/cavities were noted inside the pores of PE discs (Figure 13A,B). However, the coated PE group had a more obvious amount of collagen matrix than the non-coated PE group using Masson’s trichrome staining. Moreover, the cell population inside the pores was mostly composed of osteoblastic and osteoclast-like cells (Figure 13C,D). Remnants of coating cement, most probably HA crystals, were also noted inside the pores (Figure 13C,D). Interestingly, the connective tissue inside the pores was very vascular. This important finding was critical for bone regeneration and osteoconduction (Figure 13A,B). The coated PE showed more mature bone inside the pore than the plain PE. This could be due to the presence of bone cement inside the pore, which augmented the osteoconductive process.

The histomorphometric analysis of the quantity of bone formation in the surgical sites (expressed as a percentage) was compared to the percentage of bone in the at the native calvarias bone (Figure 13). This matched the clinical impression in that most of the bone was present in the proximal part of the surgical site (65%, SD 9.6). The more amount of bone formation was in the middle third (38%, SD 10.2).

## 3. Materials and Methods

### 3.1. Materials

The material used in the present study is porous hydrophilic PE (XM-1843 supplied by Porex Technologies GmbH, Aachen, Germany), which is supplied in the form of thin sheets (1000 × 1000 × 5 mm). According to the manufacturer’s information, the average pore size ranges from 100 to 400 μm, the pore volume is >35%, and the density is 0.46–0.54 g/cm^3^. The calcium sulfate hydroxyapatite coating was used as a thin layer on the outer surfaces of PE.

### 3.2. Characterization

The porosity of PE was observed using a scanning electron microscope (FE-SEM-JEOL JSM-6610LV- Dearborn Road, Peabody, MA, USA) operating at 5 kV. Before observation, the porous PE specimens were coated with platinum using an auto fine coater (JEOL JFC-1600, Dearborn Road, Peabody, MA, USA).

The thermal behavior (melting temperature, TGA analysis) of porous PE specimens was examined using a differential scanning calorimetry (DSC) and thermogravimetric analysis (TGA) setup type (SDT Q600, TA Instruments, New Castle, DE, USA). The specimens were placed in an aluminum pan inside the heating unit and heated at three heating rates (5, 10, and 15 °C/min) from 30 to 500 °C under nitrogen cooling. The specimen data were calculated from the DSC curves using the machine’s software. The melting temperature of the material was considered as the maximum point of the endothermic peak in the melting mode.

The creep recovery and relaxation behavior of porous PE specimens was examined using a dynamic mechanical analysis machine (RSA G2, TA Instruments, New Castle, DE, USA). The maximum load was set at 35 N, and the 3-point bending mode was used for all tests. The creep recovery behavior was examined at loads of 2, 4, and 8 N, and the temperatures used were 25, 37, 50, and 65 °C. The machine was also used for measuring the relaxation behavior of the tested materials at different strains.

The tensile behavior of porous PE was examined using a universal tensile testing machine (Jinan Testing Equipment, Century Fortune Plaza, High Tech. Development Zone, Jinan 250101, China) with a 1-kN load cell. The tests were performed at different cross-head speeds of 0.5, 1, and 2 mm/min at lab temperature. The nominal stress/strain data were estimated using the machine’s software. The ultimate strength was estimated from the maximum load, the modulus of elasticity was calculated from the initial linear portion of the data, and the fracture strain was calculated at the fracture point. The estimated data were calculated from the mean of three test results.

### 3.3. Cell Study-Related Methods

In this study, we used immortalized human bone marrow stromal cells (TERT-hBMSCs) produced by the forced overexpression of human telomerase reverse transcriptase gene in primary hBMSCs [33,34]. We used a subclone derived from TERT-hBMSCs described as CL1, which exhibits enhanced osteogenic, adipogenic, and chondrogenic differentiation potential. hBMSC-CL1 cells were cultured as previously described [35].

### 3.4. Technique Used for Coating Experimental Samples 

Porous PE scaffolds were prepared in the form of discs with an 8-mm diameter. The powdered α-calcium sulfate hydrate–hydroxyapatite (CSH–HA) was blended at a concentration of 1 g/0.43 mL of liquid. The resulting flowable slurry was used to coat the porous PE discs by dipping, shaking, blowing, and evacuation according to a previously reported protocol [36] using a dipping technique recommended to coat complex structural materials to obtain a thickness of ≤0.5 mm.

Porous PE scaffolds coated with CS–HA for better cell attachment and osteoblast differentiation were analyzed using SEM by embedding the samples into a metallurgical resin to quantify the percentage of porosity and the size of the micro-pores. Before we examined the samples, they were sputter-coated with gold. Subsequently, a Carl Zeiss (Oberkochen, Germany) Sigma VP Oxford Micro-analysis S800 or S4700 field emission SEM was used at an accelerating voltage of 10 kV.

### 3.5. Stromal Cell Attachment and Morphology Assessment

Three days after adding CL1 stromal cells to the scaffolds, cell attachment was observed using SEM. For this, CL1 cells were added to two different scaffolds with or without HA at day 0. Scaffolds were soaked overnight in fetal calf serum to improve the attachment of cells. On the day of cell addition, the scaffolds were washed once with growth media, after which 1 × 10^5^ cells were added to each scaffold. On day three, each scaffold was washed with phosphate-buffered solution and fixed with 1% glutaraldehyde (Sigma-Aldrich, St. Louis, MO, USA) buffered in 0.1 M sodium cacodylate (Agar Scientific, Stansted, UK) at 4–6 °C for SEM sample preparation. After glutaraldehyde fixation, the cells were again fixed in osmium tetroxide (1%) (Agar Scientific, Stansted, UK) according to the manufacturer’s instructions. The dry specimens were sputter-coated with gold and examined with a Carl Zeiss Sigma VP Oxford Micro-analysis S800 as reported elsewhere [30]. The scaffolds were subsequently observed and images were taken using a JSM-6360 LV SEM.

### 3.6. Cell Viability Assay by alamarBlue^®^

The cell viability of the CL1 cells grown on porous PE scaffolds was assessed with an alamarBlue^®^ cell viability assay performed according to the manufacturer’s recommendations (AbD Serotec, Raleigh, NC, USA). Briefly, 10 μL of alamarBlue^®^ substrate was added directly to the cultured cells. The cells were cultured in a 96-well plate after adding alamarBlue^®^ and incubated at 37 °C for 1 h in darkness. Fluorescence was measured with an Ex 530 nm/Em 590 nm using a BioTek Synergy II plate reader (BioTek Inc., Winooski, VT, USA).

### 3.7. In Vivo Bone Formation

This study was conducted with the approval from the ethical committee of the Dental Collage Research Centre, Dental faculty, King Saud University, Saudi Arabia. Twenty male inbred transgenic Sprague-Dawley rats (250–450 g) were obtained from the College of Food and Agriculture Sciences and kept in a dedicated animal research facility under veterinary supervision. The surgery was usually carried out after allowing for acclimatization at least two weeks from the day of arrival.

Ketamine (50 mg/kg), xylazine (6 mg/kg), and acepromazine (1 mg/kg) were intraperitoneally injected to anesthetize the animals before surgery. At the rat calvarium, the hair was shafted and skin scrubbed using Betadine (4% povidone-iodine, McKesson, San Francisco, CA, USA). We injected 1 mL of local anesthetic (Xylocaine Dental with adrenaline, 20 mg/mL + 12 µg/mL) in this area to achieve hemostasis. A 15-mm midline sagittal incision was created over the cranium, and the periosteum was carefully elevated. A circular full-thickness bone defect was trephined in the center of the parietal bone using an 8-mm trephine bur with low-speed rotation (AEU-12C Torque Plus, Aseptico, Woodinville, WA, USA) under constant irrigation with sterile saline to prevent overheating of the bone margins. Care was taken not to injure the underlying dura mater. The defects in groups 1 (*n* = 10) and 2 (*n* = 10) were subsequently filled with coated PE discs and non-coated PE discs, respectively. The periosteum was sacrificed, and the surgical wound was subsequently closed using 3-0 Vicryl sutures in layers. A postoperative analgesic (meloxicam, a non-steroidal anti-inflammatory drug) was administered subcutaneously (0.2 mg/kg) along with a prophylactic antibiotic using an oxytetracycline solution (10%, by injection at 0.2 mL/kg). After full recovery, the rats were transferred to a normal holding cage, and 10 mL of saline were given subcutaneously to avoid dehydration. The activities of the rats were monitored daily, as was the operative site for bleeding or signs of infection. After surgery, the rats were housed in static micro-isolator cages at the Animal Research Housing facility of the King Saud University, College of Medicine. Each morning, a laboratory animal technician observed all animals for signs of illness, injury, infection, or death. Harlan rodent diet 20202X was offered to the animals and all water bottles were refilled daily. Bedding was changed weekly after spraying rocks with rescue RTU and cleaning the cages.

All animals completed the full follow-up period (8 weeks) with no complications. Animals were euthanized 8 weeks after craniotomy using an overdose of sodium pentobarbital (140 mg/kg, injected subcutaneously). The cranial defect sites were harvested along with the surrounding bones. The harvested samples were fixed in 10% formalin for subsequent radiographic and histology assessments.

### 3.8. Radiographic Assessment

#### Cone Beam Tomography

Images were obtained and analyzed using Planmeca ProMax^®^ 3D Classic (Planmeca, Helsinki, Finland) with settings of 120 kV, 5 mA, 18.54 mAs, resolution of 0.4 mm pixel/voxel, and field size of 2.0 mm, as previously described [37]. The harvested cranial defect sites were kept on a stable mounting table in aqueous medium during image capture to improve the captured image contrast of the soft tissue [38]. Data were stored on optical discs to assess the cross-sectional area of the bone tissue. The main aim of performing bone scans was to detect any bone regeneration at the scaffolds and bone interface or/and inside the pores.

### 3.9. Histological Analysis 

#### Slide Preparation

Samples were transferred to plastic containers containing 10% buffered formalin (*w*/*v*). The cuts divided the regenerated tissue and its surrounding native bone into upper, middle, and lower sections. Specimens were removed from the 10% buffered formalin, loaded into cassettes of a suitable size, and placed in a rotor basket in buffered 10% formic acid for decalcification. Fluoroscopy was used to check the end point of the decalcification process to avoid excessive damage to the tissue. The decalcified tissue blocks were embedded in paraffin wax and 5-µm sections were prepared. The sections were subsequently stained with hematoxylin and eosin and Masson’s trichrome stain and mounted on histological glass slides prior to assessment. The slides were subsequently examined under light microscopy (Zeiss, Oberkochen, Germany). Representative areas were captured using different objectives (5×, 10×, 20×, and 40×) using an AxioVision camera (Carl Zeiss Microscopy GmbH, Jena, Germany), and images were saved as TIFF files.

Bone regeneration, quality, and graft incorporation were assessed. The area of scaffold and native bone interface was assessed in both experimental groups. Furthermore, the pores of the implanted scaffold were observed in the different study groups. Remnants of the coated bone cement on PE discs were also examined. The nature of cell populations around and inside the PE disc was evaluated. On another hand quantitative data was obtained using histomorphometery to estimate the percentage of regenerated bone, residual cement (empty space), and fibrous/muscular tissue was estimated, following well established protocol [32].

## 4. Conclusions

In this study, porous PE scaffolds were examined as potential bone substitutes. The results showed that the material has well-interconnected pores that are suitable for cell growth. The creep recovery, relaxation, and tensile test results showed that the material has suitable mechanical strength to support bone ingrowth. The material is applicable in stress-bearing areas in clinical practice. This study clearly demonstrated the role of human bone marrow mesenchymal cells in tissue engineering. The material was porous, which allowed it to be covered with CS–HA cement in the pores. Furthermore, hBMSCs attached with higher affinity to the coated PE than to the non-coated PE. The hBMSCs demonstrated significantly higher proliferation on porous PE scaffolds coated with CS–HA, indicating that CS–HA is an important factor for the attachment and growth of hBMSCs on porous PE scaffolds that might play an essential role in bone formation in vivo. The in vivo tests showed that both study groups experienced active bone remodeling at the border of the defect. Bone regeneration at the border was also evident, which confirmed that PE (coated or non-coated) acted as an osteoconductive bone graft. Furthermore, bone formation was present inside the pores in both groups. However, the presence of remnant bone cement (CS–HA) in coated PE scaffolds accelerated the process, and the presence of collagen and mature osteoid was pronounced in the coated PE discs. This study provided evidence that coated PE materials have clear clinical potential because they facilitate bone regeneration, enhance the early osteointegration of graft materials, and therefore minimize graft migration and infection.

## Figures and Tables

**Table 1:** Stress–strain data for porous polyethylene at different cross-head speeds.

Cross-Head Speed	Young’s Modulus (MPa)	Ultimate Strength (MPa)	Fracture Strain %
0.5 mm/min	480 ± 16	2.1 ± 0.1	68 ± 3
1 mm/min	560 ± 20	2.9 ± 0.15	56 ± 2
2 mm/min	580 ± 12	3.8 ± 0.13	50 ± 4
